# The effects of induced hindgut acidosis in sheep on rumen fermentation and gut permeability

**DOI:** 10.1093/jas/skaf289

**Published:** 2025-08-27

**Authors:** Haley F Linder, Emma G Prybylski, Brett R Loman, Santiago D Gutierrez-Nibeyro, Edgar F Garrett, Joshua C McCann

**Affiliations:** Department of Animal Sciences, University of Illinois Urbana-Champaign, Urbana, IL, USA61801; Department of Animal Sciences, University of Illinois Urbana-Champaign, Urbana, IL, USA61801; Department of Animal Sciences, University of Illinois Urbana-Champaign, Urbana, IL, USA61801; College of Veterinary Medicine, University of Illinois Urbana-Champaign, Urbana, IL, USA61801; College of Veterinary Medicine, University of Illinois Urbana-Champaign, Urbana, IL, USA61801; Department of Animal Sciences, University of Illinois Urbana-Champaign, Urbana, IL, USA61801

**Keywords:** hindgut acidosis, leaky gut

## Abstract

The objective was to determine the effects of induced hindgut acidosis in sheep on cecal pH, ruminal fermentation, and gut permeability. Eleven ruminally and cecally cannulated ewes (49 ± 4 kg) were assigned to one of two treatments: control (CON; *n* = 5) or induced hindgut acidosis (HGA; *n* = 6). To induce hindgut acidosis, 3 g wheat starch/kg BW per 24 h was continuously infused via the cecal cannula for 4 d. Control ewes received a constant infusion of deionized water. Ewes were fed a common diet at a set level of intake based on body weight. Chromium EDTA was dosed once daily via the cecal cannula as a marker of gut permeability. Rumen, cecal, and fecal samples were collected to determine pH and volatile fatty acids (VFA). Rumen fluid was collected on day 4 for an ex vivo fermentation to determine pH, VFA, ammonia, and in vitro dry matter digestibility (IVDMD). On day 5, sucralose was infused through the cecal cannula and blood was collected from a mesenteric catheter under anesthesia. Transepithelial electrical resistance (TEER) was determined in the ileum, cecum, and colon in Ussing chambers. There was a treatment × time effect (*P* = 0.05) for cecal pH, with HGA ewes having lesser cecal pH after day 1. By day 4, cecal pH had dropped to 5.07 for HGA ewes compared to 6.40 for CON ewes. A treatment × time interaction was observed (*P* < 0.01) for fecal pH and followed the same trend as cecal pH. Total fecal VFA concentration was greater (*P* < 0.01) in HGA ewes than CON. Rumen pH was not affected (*P* = 0.87) by the interaction of treatment × time, but was affected (*P* < 0.01) by treatment, as ewes on the HGA treatment had a lesser rumen pH than CON ewes. Control ewes had lesser ruminal VFA and ammonia concentrations than HGA ewes (*P* < 0.01). Despite this, the ex vivo fermentation did not indicate any differences in pH, VFA, or IVDMD (*P* ≥ 0.11). Urinary Cr recovery was not affected by the interaction of treatment × time, or treatment (*P* ≥ 0.13). There were no effects (*P* ≥ 0.22) of treatment, time, or their interaction on mesenteric plasma sucralose concentration. In cecal tissue, TEER tended (*P* = 0.09) to be lesser, indicating increased permeability in HGA ewes compared with CON ewes. In contrast, TEER was not different (*P* ≥ 0.83) in ileal or colonic tissues between treatment groups. A cecal infusion of starch induced hindgut acidosis and affected hindgut fermentation. Hindgut acidosis still had systemic effects on rumen conditions despite varied responses in gut permeability.

## Teaser

A novel model of ruminally and cecally cannulated ewes was utilized to determine the effects of induced hindgut acidosis on rumen fermentation and gut permeability.

## Introduction

Hindgut fermentation can contribute 5% to 10% of dietary energy in ruminants (Gressley et al., 2011). The cecum in ruminant animals is often overlooked, as the rumen is the primary and initial site of microbial fermentation. However, increased flow of substrates to the hindgut can increase fermentation (Siciliano-Jones and Murphy, 1989). Nonstructural carbohydrates (**NSC**) are fed to feedlot cattle to meet production demands as they are more cost-effective per unit of gain than forages. Greater inclusion of NSC in diets results in a decrease in rumen starch digestibility and an increase in ruminal passage rate (Krizsan et al., 2010; Moharrery et al., 2014). Considering only 35% to 60% of starch is digested in the ruminant small intestine (Harmon, 2009), greater starch flow to the hindgut would increase fermentation and organic acid accumulation in the hindgut. A subsequent decline in intraluminal pH can result in hindgut acidosis. However, the hindgut differs in epithelial structure and buffering capacity from the rumen. The protective mucus and single layer of columnar epithelium of the cecum are more sensitive to sudden pH changes than the stratified squamous lining of the rumen (Steele et al., 2016). Additionally, the lack of salivary bicarbonate as a buffer source can further exacerbate the severity of acidic conditions in the hindgut (Gressley et al., 2011). Exposure to acidic conditions may damage hindgut epithelium and increase gut permeability (Sanz-Fernandez et al., 2020).

The intestinal epithelium maintains a barrier between the luminal contents of the gut and the systemic circulation. Increased gut permeability allows for a flux of unwanted luminal contents, such as bacteria and their associated compounds, into circulation (Mani et al., 2012). Disruptions in gut barrier function can result in inflammation and reduced dry matter (DM) intake in cattle (Kvidera et al., 2017). Systemic inflammation resulting from hindgut barrier disruption may also influence rumen fermentation, as inflammatory stimuli such as lipopolysaccharide have been shown to alter ruminal microbial communities and disrupt ruminal metabolism (Jing et al., 2014). Additionally, hindgut acidosis can decrease total tract digestibility of nutrients (van Gastelen et al., 2021a, b). To date, limited research has investigated the ruminant animal response to a hindgut acidosis challenge. Furthermore, the potential effects of hindgut acidosis on rumen fermentation parameters have yet to be defined. A novel ruminally and cecally cannulated sheep model was utilized to directly induce acidosis in the cecum. Therefore, the objective was to determine the effects of induced hindgut acidosis in sheep on cecal pH, ruminal fermentation, and gut permeability.

## Materials and Methods

**Figure 1.**
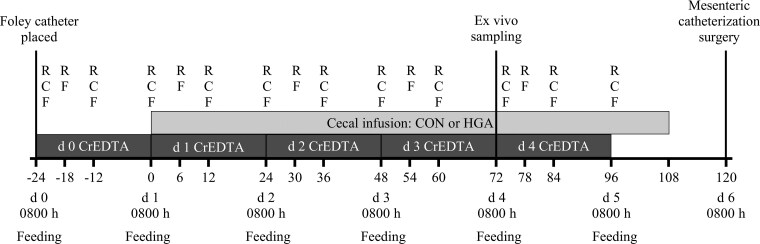
Experimental timeline to determine the effects of induced hindgut acidosis in sheep on cecal pH, ruminal fermentation, and gut permeability. Continuous cecal infusion treatments were applied from day 1 to 4 and included control (CON; 26 mL H_2_O/kg BW per 24 h) or induced hindgut acidosis (HGA; 3 g wheat starch + 26 mL H_2_O/kg BW per 24 h). A pulse dose of CrEDTA was given daily in the cecal cannula at 0800 hours, with total urine collected over the subsequent 24 h. Daily sampling of contents was conducted from the rumen (R), cecum (C), and feces (F).

The experimental protocol was approved by the Institutional Animal Care and Use Committee at the University of Illinois at Urbana-Champaign (Protocol # 22078). Eleven ruminally and cecally cannulated Katahdin and Katahdin-cross ewes (body weight [BW] = 49 ± 4 kg) were utilized in three blocks to determine the contribution of induced hindgut acidosis on ruminal pH and fermentation and gut permeability. Sheep were selected as the model due to the terminal nature of the study. The cecal cannula was a T-shaped titanium cannula (internal diameter = 2 cm) as described in Stein et al. (1998) and placed in the body of the cecum. Ewes recovered a minimum of two weeks before initiation of the experiment. An experimental timeline is presented in Figure 1. During the experiment, sheep were housed in a climate-controlled metabolism barn, set to 18.3 °C, at the University of Illinois Beef Cattle and Sheep Field Research Laboratory in Urbana, IL. For the 5 d experimental period, ewes were housed in individual stainless steel metabolism crates (1.5 m × 0.9 m × 1.2 m) that contained two 7.6-L plastic buckets for feed and water. Sheep were acclimated to the metabolism crate prior to the study through short-term exposure over a period of several weeks. Sheep were then moved into crates 72 h before the start of infusions to allow for full readjustment and minimize any effects on intake and behavior. While in metabolism crates, ewes were fitted with a nylon halter and tethered to the side of the crate to prevent disturbing the cecal infusion lines.

### Metabolism experiment

Prior to the experimental treatments being applied, fecal pH was collected as explained in detail in a later section for 2 consecutive days, averaged, and then used to stratify ewes within the block. After stratification, 1 of the 2 treatments was then randomly assigned: control (**CON**) or induced hindgut acidosis (**HGA**). Control ewes received a continuous cecal infusion of deionized water (26 mL/kg BW per 24 h). Ewes on the HGA treatment received a continuous cecal infusion of a starch solution (3 g wheat starch and 26 mL H_2_O/kg BW per 24 h) to induce hindgut acidosis. Starch dosage was selected based on multiple abomasal starch infusion studies in cattle, and one study with a cecal infusion of starch in sheep (Ørskov et al., 1970; Gressley et al., 2011).

Initially, 6 ewes were assigned to each treatment. However, due to health complications unrelated to experimental procedures, one ewe was euthanized during the trial, resulting in five ewes in the control treatment. Wheat starch (Aytex-P; Archer Daniels Midland Company, Chicago, IL, USA) was made into a suspension with deionized water (8.7 mL/g wheat starch) and continuously mixed via magnetic stir plate. Solutions were prepared fresh every 12 h for use over 12 h intervals. Both treatments were infused through Tygon tubing (internal diameter = 3.18 mm; Saint-Gobain North America, Valley Forge, PA, USA) with a peristaltic pump (Masterflex L/S; Cole-Parmer Instrument Company, Vernon Hills, IL, USA). Rates varied depending on the volume to be infused over 12 h intervals. For HGA ewes, tubing was flushed with deionized water every 12 h to prevent accumulation of any residual starch solution. Infusions started at 0800 hours on day 1 of the experimental period and were stopped at 2000 hours on day 5.

Sheep were dosed with 200 mL of CrEDTA (554 mg Cr), prepared according to Binnerts et al. (1968) in the cecal cannula at 0800 hours on days 1 to 4. Additionally, a dose of CrEDTA was given 24 h before the start of cecal infusions (day 0) to establish a baseline value of urinary CrEDTA excretion for each ewe, since even in healthy ruminant animals, 2% to 3% of ruminally dosed CrEDTA can be recovered in urine (Udén et al., 1980). To collect urine, on day 0, ewes were fitted with a 10 Fr silicone Foley urinary catheter (MILA International, Inc., Florence, KY, USA). The urinary catheter was attached via collection line to a container outside the metabolism crate. Total urine volume over 24 h was measured, and a 15 mL aliquot was taken on days 0, 1, 2, 3, and 4. Urine was frozen at −20 °C for subsequent analysis of Cr.

Sheep were fed once daily at 0800 hours and offered 1.8% of BW on a DM basis on days 0, 1, 2, and 1.1% on days 3, 4, and 5. Feed allocation was reduced on days 3, 4, and 5 for both treatments to more closely align with voluntary intake levels of HGA sheep during the challenge. To reduce the potential effects of intake on gut permeability, if sheep did not consume their allotted feed amount by 2000 hours, the remainder was dosed via the rumen cannula. The diet (Table 1) consisted of 40% grass hay pellets, 35% whole shelled corn, 15% dried distillers’ grains, and 10% alfalfa cubes (DM basis). Feed ingredient samples were taken at the start of the experiment and stored at −20˚C for later analysis. Animals had ad libitum access to water and a mineral block (Agrimaster Sheep and Goat Mineral Block; Blain Supply Inc., Janesville, WI, USA) containing 45% NaCl; 7% Ca, 5% P; 1% Mg; 5,000 ppm Fe; 150 ppm Co; 150 ppm Zn; 125 ppm I; 10 ppm Se. Feces were collected on days 2 to 4 via stainless steel pans under each metabolism crate, weighed, and stored at −20 ˚C until further analysis.

**Table 1.** In vivo diet and ex vivo substrate composition and chemical analysis

Ingredient, % DM	
Grass hay pellets	40.0
Whole shell corn	35.0
Dried distillers grains	15.0
Alfalfa cubes	10.0
Chemical analysis, % DM	
Dry matter	90.5
Organic matter	93.9
Crude protein	14.4
Neutral detergent fiber	39.7
Acid detergent fiber	22.7
Starch	26.4
Ether extract	3.7

Rumen contents were collected via rumen cannula and strained through 2 layers of cheesecloth for rumen fluid collection. Samples were taken immediately before feeding, and 6 and 12 h post-feeding on days 0 and 1 to 4 to measure rumen pH with a benchtop pH meter (Accumet Basic AB15; Fisher Scientific, Hampton, NH, USA). For volatile fatty acids (VFA) analysis, 4 mL of rumen fluid was added to a tube containing 4 mL of 2N HCL, and for NH_3_ analysis, 8 mL of rumen fluid was added to a tube containing 2 mL of 2N HCL. All tubes were immediately frozen and stored at −20 °C until laboratory analysis. A 5 g fecal grab sample was also collected and mixed with 5 ml of distilled water to measure fecal pH at the same time points as rumen fluid. Cecal fluid was collected via cecal cannula immediately before feeding, and 12 h post-feeding on days 0, 1 to 4 for pH measurement. On days 0, 2, and 4, a grab sample of feces was sampled immediately before feeding and 12 h later, snap frozen in 2 mL cyrovials in liquid nitrogen, kept on ice, and stored at −80 °C for later VFA analysis.

On day 5, sheep were offered 1.1% of their BW of the diet from 0800 to 2000 hours. Feed was then removed, and animals fasted in preparation for surgery the following day. Cecal infusions ceased at 2000 hours on day 5 as well. Sheep were transported to the University of Illinois College of Veterinary Medicine Large Animal Clinic at approximately 0600 hours on day 6 for mesenteric catheterization surgery. Surgery was conducted at the University of Illinois College of Veterinary Medicine Large Animal Clinic. Animals were anesthetized with a combination of midazolam (0.1 to 0.2 mg/kg IV) and ketamine (2 to 4 mg/kg IV) with isoflurane inhalant anesthetic to maintain general anesthesia. For mesenteric vein catheterization, the sheep were in left lateral recumbency. A 25 to 35 cm paracostal incision was made 5 cm caudal to the last rib and continued through the subcutaneous, muscle layers, and the peritoneum. The abdominal viscera were packed off with saline-moistened laparotomy sponges. A mesenteric vein was identified near the caudal end of the cecum and isolated by blunt dissection. A 20G IV catheter (BD Insyte; Becton, Dickinson and Company, Franklin Lakes, NJ, USA) that had been treated with 2% TDMAC-heparin complex was inserted approximately 15 cm into the mesenteric vein, then sutured into place with 2 to 0 VICRYL suture (Ethicon Inc., Raritan, NJ, USA). An extension set was then attached to the catheter for ease of collection.

After catheters were in place, a priming dose of 15 mL of 7% wt/vol paraaminohippurate (**PAH**) was infused through the mesenteric catheter as a measure of mesenteric blood flow rate. Throughout the duration of the surgery, PAH was infused at a rate of 1 mL/min. Paraaminohippurate was prepared according to Huntington et al. (1989).

After the priming dose of PAH was infused, a solution of 1 g of sucralose (Thermo Fisher Scientific, Waltham, MA, USA) dissolved in 60 mL of deionized water was infused through the cecal cannula as a pulse dose to evaluate the permeability of the large intestine. Approximately 5 mL of blood was collected from the mesenteric venous catheter at hours 0, 0.5, 1, 2, 3, and 4, post sugar marker infusion. Additionally, approximately 5 mL of blood was collected from the hepatic venous catheter at hours 1, 2, 3, and 4, post sugar marker infusion. Blood was collected in K_2_EDTA tubes (BD Vacutainer; Becton Dickinson and Company, Franklin Lakes, NJ, USA) and centrifuged at 4,500 × *g* for 20 min, and plasma was collected. Plasma was stored in 2 mL tubes and frozen at − 20 °C until analysis.

Feed ingredient samples and feces were dried in a 55 °C oven and ground through a Wiley mill (1-mm screen, Arthur H. Thomas, Philadelphia, PA, USA). Samples were analyzed for DM (24 h at 105 °C), neutral detergent fiber (**NDF**) and acid detergent fiber (using Ankom Technology method 5 and 6, respectively; Ankom^200^ Fiber Analyzer, Ankom Technology, Macedon, NY, USA), nitrogen by combustion to estimate crude protein (Leco TruMac; LECO Corporation, St. Joseph, MI, USA), ether extract (Ankom method 2; Ankom Technology), and organic matter (600 °C for 12 h; Thermolyte muffle oven Model F30420C; Thermo Scientific, Waltham, MA, USA). Fecal water loss was calculated as the difference between as-is fecal weight and dry fecal weight. Starch analysis was conducted at DairyLand Laboratories Inc. (Arcadia, WI, USA) using a YSI 2700 Select Biochemistry analyzer (AOAC Official Method 2014.10; AOAC International, 2019; Yellow Springs Instrument Inc., Yellow Springs, OH, USA).

Rumen fluid samples were thawed and analyzed for ammonia concentrations with the method described by Broderick and Kang (1980) using a spectrophotometer (SpectraMax QuickDrop; Molecular Devices, San Jose, CA, USA). Ruminal and fecal concentrations of VFA were determined by gas chromatography (HP5890 series, Hewlett-Packard, Wilmington, DE, USA) on a glass column using an adapted method from Erwin et al. (1961).

Chromium analysis was conducted at Analab (Fulton, IL, USA). Samples were diluted 1:20 in 1% nitric acid, passed through a 0.45 micron filter, then analyzed by inductively coupled plasma instrumentation (Agilent 5100 ICP-OES, Agilent Technologies, Santa Clara, CA, USA) with AOAC method 985.01, but without additional chemical or temperature-induced digestion. Urinary Cr content at each time point was calculated as urine volume × Cr concentration.

Sucralose and PAH plasma concentrations were analyzed at the Carver Metabolomics Core, University of Illinois Urbana-Champaign Roy. J. Carver Biotechnology Center. For sucralose quantification, plasma samples (25 μL) were spiked with 5 μL of internal standard (sucralose-d6, 10 μg/mL) and 70 μL of pure methanol, vortexed, and centrifuged for 10 min at 18,000 rpm. Supernatant was collected, and 1 μL was injected into the LC-MS system, described below. For PAH quantification, plasma samples were diluted 250 times, spiked with 5uL of its internal standard (4-aminohippuric-d4 acid, 1 ug/mL) and 1 uL was injected into the LCMS system, which consisted of a 5500 QTRAP mass spectrometer (SCIEX, Redwood City, CA, USA) and an Agilent 1200 HPLC system (Agilent Technologies, Santa Clara, CA, USA) equipped with a Thermo Hypersil GOLD column (2.1 × 150 mm, 1.9 μm; Thermo Fisher Scientific, Waltham, MA, USA). Mobile phase A was 0.1% formic acid in water, and mobile phase B was 0.1% formic acid in acetonitrile. The flow rate was 0.7 mL/min, and the column temperature was 60 °C. Mass spectra were acquired under negative ESI with the ion spray voltage at −4,500 V. The source temperature was 450 °C. The curtain gas, ion source gas 1, and ion source gas 2 were 13.8, 413.7, and 379.2 kPa, respectively.

Mesenteric blood flow was calculated based on Huntington et al. (1989):


$$\begin{array}{l} Blood\;f\;\;\;low\;\left(mL/min\right)=\frac{IR}{M-H\;} \end{array}$$

where IR is the infusion rate of PAH (mg/mL) and M and H are the PAH concentration (mg/mL) in mesenteric and hepatic blood, respectively.

The MIXED procedure of SAS 9.4 (SAS Inst. Inc., Cary, NC) was used for all statistical analyses. Repeated measures were used for rumen pH, cecal pH, fecal pH, ruminal ammonia and VFA concentration, urinary Cr content, mesenteric sucralose plasma concentration, and blood flow rate with fixed effects of treatment, time, and the interaction of treatment × time. The covariance structure was first-order autoregressive, based on the lowest Akaike Information Criteria. If applicable, values from day 0 were averaged across all time points and included in the model as a covariate. Significance was declared at *P* ≤ 0.05, and tendencies were discussed at 0.05 < *P* ≤ 0.10.

### *Rumen* ex vivo *fermentation*

An ex vivo fermentation was also conducted to further investigate the effects of hindgut acidosis induction on rumen fermentation. The terminology ex vivo is utilized here to distinguish this assay from a conventional in vitro fermentation, as treatments were applied directly to the animal and not to the fermentation flask. As such, the rumen fluid carried the in vivo treatment effects at the time of collection, and the animal remained the experimental unit. The ex vivo fermentation was conducted concurrently with the metabolism experiment and utilized the same ewes, treatments, and feeding strategies as previously described. Approximately 300 mL of rumen fluid was collected from each ewe immediately before feeding on days 0 and 4 of the metabolism experiment. To collect rumen fluid, rumen contents were collected via rumen cannula and strained through two layers of cheesecloth. Rumen fluid was added to two 250 mL Erlenmeyer flasks. Flasks contained 2.5 g of substrate and 50 mL of rumen fluid, and 100 mL of McDougall’s artificial saliva (McDougall, 1948). Four sealed ANKOM F57 bags were filled with 0.25 g DM of substrate and placed into flasks to determine in vitro DM digestibility (**IVDMD)**. The substrate was ground (2 mm) and composed of the same ingredients as the experimental diet (40% grass hay pellet, 35% whole shell corn, 15% dried distillers’ grain, and 10% alfalfa cubes). Each flask was purged with CO_2_ and fitted with a rubber stopper with a one-way air valve and sampling line. Flasks were incubated in a 39 °C water bath (Isotemp SWB27; ThermoFisher Scientific, Waltham, MA, USA) and shaken at 130 rpm for 24 h. At the conclusion of the ex vivo fermentation, ANKOM bags were removed from flasks, rinsed with deionized water, dried at 55 °C for 12 h, and then weighed for IVDMD determination. Sample aliquots were collected at hours 8 and 24 to determine pH, VFA, and ammonia utilizing the same procedures as previously described. Data were analyzed using the MIXED procedure of SAS 9.4. For all response variables, values from the run before cecal infusion initiation (day 0) were included in the model as a covariate. Ammonia, pH, and VFA were analyzed with the effects of treatment, time, and their interaction.

### Ussing chamber measurements

Following the conclusion of the intestinal catheterization surgery, while still anesthetized, sheep were euthanized with pentobarbital (85 mg/kg IV). Immediately following euthanasia, cecal, ileum, and spiral colon tissue samples were collected. Cecal tissue samples were taken from the midbody region of the cecum, opposite the side of the cannula. Ileal samples were collected approximately 20 cm from the ileocecal junction, and spiral colon tissue samples were collected from the second centripetal loop of the spiral colon, approximately 30 to 40 cm distal to the cecocolic junction. Lumen contents of all tissue samples were flushed with Ringer’s solution before transfer to specimen jars with Krebs bicarbonate Ringer’s solution, prepared according to Clarke (2009). Samples were transported on ice to a laboratory and utilized within two hours of collection.

In the laboratory, tissue was prepared similarly to Clarke (2009). Briefly, intestinal tissues were opened longitudinally along the mesenteric attachment remnant, then a smaller section of the antimesenteric mucosa was cut. Sections with visible lesions were avoided in order to best measure paracellular movement and decrease variability (Clarke, 2009; Pederzolli et al., 2018). The serosa, longitudinal, and circular muscle layers were stripped using a clean microscope slide, leaving the underlying submucosal elements and epithelium. Mucosa was cut and mounted on pins of tissue sliders of a Ussing chamber. The exposed surface area of the mucosa was 0.3 cm^2^. One piece of tissue per site per animal was utilized in the Ussing chambers.

Krebs bicarbonate Ringer’s solution, maintained at 37 °C and a pH of 7.4, was on both sides of the intestinal tissue in the Ussing chamber. Carbogen gas (95% oxygen, 5% carbon dioxide) oxygenated the buffer on both sides of the chamber. All tissues were incubated under short-circuit conditions using a computer-controlled voltage clamp device (Physiologic Instruments, Reno, NV, USA). Potential difference across the epithelia was measured by electrodes (Physiologic Instruments, Reno, NV, USA) connected to the Ussing chamber by agar bridges (3% agar in 3 M KCl). Tissues were equilibrated for 20 min then transepithelial electrical resistance (**TEER**) in ohms was recorded for 10 min and used for statistical analysis (Clarke, 2009). The MIXED procedure of SAS 9.4 (SAS Inst. Inc., Cary, NC) was utilized with the fixed effect of treatment.

## Results

### Metabolism experiment

**Figure 2.**
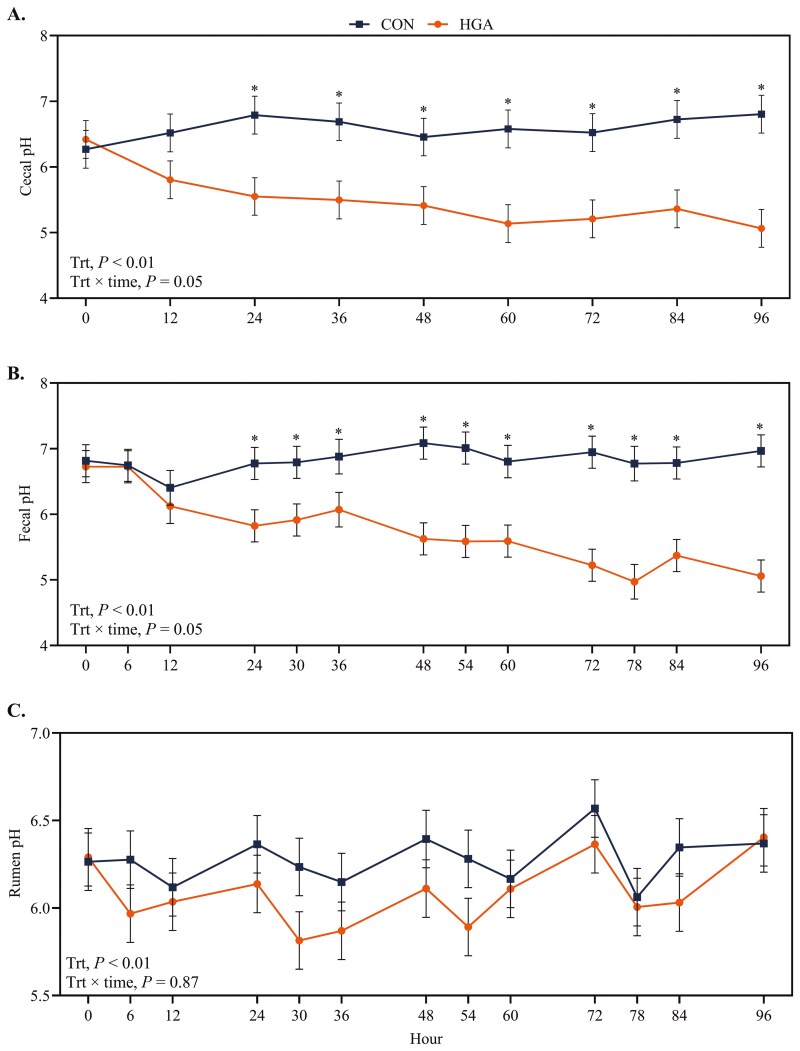
Effect of induced hindgut acidosis in ewes on cecal pH (A), fecal pH (B), and rumen pH (C). CON = control, 26 mL H_2_O/kg BW per 24 h; HGA = induced hindgut acidosis, 3 g wheat starch + 26 mL H_2_O/kg BW per 24 h. If a treatment × time effect was observed, pairwise treatment differences were sliced by time and indicated by * when *P* ≤ 0.05.

There was a treatment × time effect (*P *= 0.05) for cecal pH, with HGA ewes having a lesser cecal pH after day 1 (Figure 2). A treatment × time interaction was also observed (*P *< 0.01) for fecal pH and followed the same trend as cecal pH. Total fecal VFA concentration was not affected (*P *= 0.26) by the interaction of treatment × time (Table 2). However, it was affected (*P < *0.01) by treatment as HGA ewes had greater fecal total VFA concentrations than CON ewes, 97.7 versus 47.7 µmol/g DM, respectively. Butyrate molar percentage was greater (*P *< 0.01) in HGA than CON ewes, 10.4% versus 4.0%, respectively. There were no effects (*P ≥ *0.22) of treatment, time, or their interaction on the molar percentage of other VFA. Daily fecal output was only impacted by treatment, with fecal output being greater (*P *= 0.02) in HGA than CON ewes (Table 3). Fecal DM percentage was lesser (*P *< 0.01) in HGA ewes than in CON ewes. Fecal water loss was greater (*P *< 0.01) in HGA ewes than in CON ewes. As expected, HGA ewes had greater fecal starch (*P *< 0.01) than CON ewes, 3.5% versus 0.2%, respectively.

**Table 2.** Effect of induced hindgut acidosis in ewes on fecal VFA concentration, and ruminal VFA and ammonia concentration

	Treatment^1^			*P*-value^2^		
Item	CON	HGA	SEM	Trt	Time	Trt × time
**Fecal**						
Total VFA, µmol/g DM	47.7	97.7	10.27	<0.01	0.46	0.26
VFA, % total						
Acetate	76.4	72.5	2.46	0.28	0.69	0.64
Propionate	14.6	11.3	1.77	0.20	0.68	0.85
Butyrate	4.0	10.4	1.16	<0.01	0.87	0.97
Valerate	1.0	1.0	0.27	0.99	0.27	0.38
Isovalerate	2.4	3.2	0.68	0.42	0.72	0.48
Isobutyrate	1.4	2.1	0.46	0.33	0.95	0.98
**Rumen**						
pH	6.28	6.08	0.07	<0.01	0.04	0.87
Total VFA, mM	53.7	62.1	2.66	<0.01	<0.01	0.99
VFA, % total mM						
Acetate	54.8	54.3	0.62	0.48	0.23	0.87
Propionate	24.5	24.5	1.15	0.96	<0.01	0.91
Butyrate	16.6	17.0	0.74	0.69	<0.01	0.26
Valerate	1.2	1.5	0.13	0.02	0.60	0.61
Isovalerate	1.7	1.7	0.11	0.62	<0.01	0.89
Isobutyrate	1.1	1.1	0.08	0.89	<0.01	0.99
Ammonia, mM	6.4	9.1	0.69	<0.01	<0.01	0.68

^1^CON = control, 26 mL H_2_O/kg BW per 24 h; HGA = induced hindgut acidosis, 3 g wheat starch + 26 mL H_2_O/kg BW per 24 h.

^2^Trt = treatment effect; trt × time = treatment × time effect.

**Table 3.** Effect of induced hindgut acidosis in ewes on fecal dry matter and starch, and urine volume

	Treatment^1^			
Item	CON	HGA	SEM	*P*-value
Feces, g/d	144	198	18.8	0.02
Fecal DM, %	49.0	26.7	2.53	<0.01
Fecal water loss, g/d	160	558	67.4	<0.01
Fecal starch, % DM	0.2	3.5	0.64	<0.01
Urine volume, mL/d	1150	852	113.5	0.02

^1^CON = control, 26 mL H_2_O/kg BW per 24 h; HGA = induced hindgut acidosis, 3 g wheat starch + 26 mL H_2_O/kg BW per 24 h.

Rumen pH was not affected by the interaction of treatment × time (*P* = 0.87), but it was affected (*P *< 0.01) by treatment as ewes on the HGA treatment had a lesser rumen pH than CON ewes, 6.08 versus 6.28, respectively. There was no treatment × time interaction (*P *= 0.68) for rumen ammonia concentration. However, control ewes did have lesser ammonia concentration than HGA ewes (*P* < 0.01; Table 2). Total ruminal VFA concentration was also affected by treatment (*P* < 0.01), with HGA ewes also having greater total VFA than CON ewes. Acetate molar proportion was not affected (*P *≥ 0.22) by treatment, time, or their interaction. Propionate, butyrate, isovalerate, and isobutyrate molar proportions were only affected by time (*P *< 0.01). Specifically, propionate increased following feeding, but butyrate, isovalerate, and isobutyrate decreased following feeding. Control ewes had lesser valerate molar proportion than HGA ewes (*P* = 0.02).

**Figure 3.**
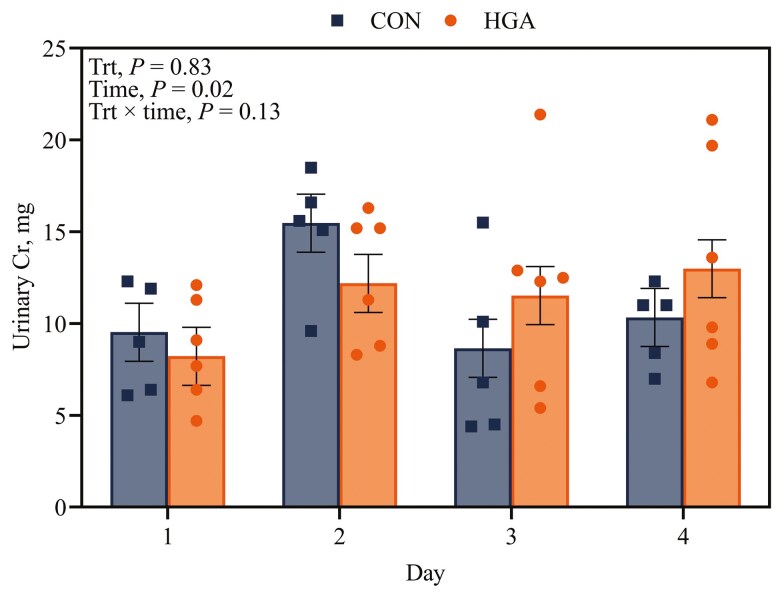
Effect of induced hindgut acidosis in ewes on urinary chromium excretion over the experimental period. CON = control, 26 mL H_2_O/kg BW per 24 h; HGA = induced hindgut acidosis, 3 g wheat starch + 26 mL H_2_O/kg BW per 24 h.

Urinary Cr recovery was not affected (*P *= 0.13) by the interaction of treatment × time (Figure 3). There was no difference between treatments for urinary Cr recovery (*P *= 0.83). However, urinary Cr recovery did differ between days (*P *= 0.02). Urine production volume was only affected by treatment (*P *= 0.02) as CON ewes had greater urine production than HGA ewes (Table 3).

**Figure 4.**
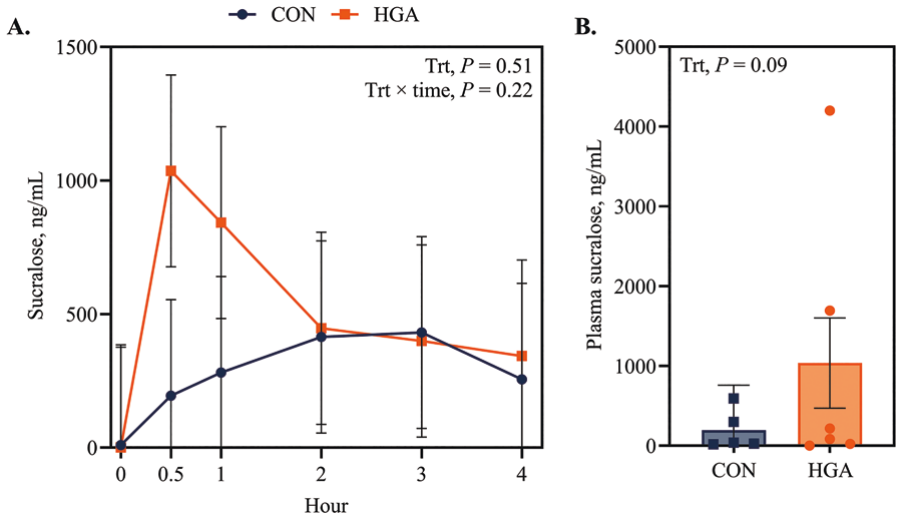
Effect of induced hindgut acidosis in ewes on mesenteric plasma sucralose concentration over time (A) and at hours 0.5 post-infusion (B). CON = control, 26 mL H_2_O/kg BW per 24 h; HGA = induced hindgut acidosis, 3 g wheat starch + 26 mL H_2_O/kg BW per 24 h.

Mesenteric blood flow was not affected (*P *≥ 0.13) by treatment, time, or their interaction. Mesenteric plasma sucralose concentration was not affected (*P *= 0.22) by the interaction of treatment × time (Figure 4). There was a tendency for a time effect (*P *= 0.07), with hour 0.5 having the greatest sucralose concentration. There were no statistical differences between treatments (*P *= 0.51). However, there was a large numerical difference observed at hour 0.5, as plasma sucralose concentration was 1036 ng/mL for HGA ewes vs 194 ng/mL for CON ewes.

### Ex vivo *fermentation*

In vitro DM disappearance on day 4 was not affected (*P *= 0.60) by cecal infusion treatment (Table 4). There was no effect (*P* = 0.78) of treatment on ex vivo pH; however, there was a time effect (*P* = 0.01) with pH decreasing over time. There were no effects (*P* ≥ 0.11) of treatment, time, or their interaction on ex vivo ammonia or total VFA concentration. Molar proportions of acetate, propionate, and butyrate were also not affected (*P *≥ 0.13) by treatment, time, or their interaction.

**Table 4.** Effect of induced hindgut acidosis in ewes on rumen ex vivo fermentation parameters on day 4

	Treatment^1^			*P*-value^2^		
Item	CON	HGA	SEM	Trt	Time	Trt × time
IVDMD^3^	60.7	59.8	1.21	0.60	-	-
pH			0.080	0.78	<0.01	0.98
8 h	6.84	6.86				
24 h	6.47	6.50				
Ammonia, mM	9.5	13.1	1.46	0.11	0.31	0.33
Total VFA, mM	49.8	50.3	2.65	0.89	0.18	0.42
VFA, % total mM						
Acetate	48.1	54.3	0.36	0.24	0.33	0.32
Propionate	29.9	27.7	0.21	0.46	0.13	0.27
Butyrate	16.0	14.6	0.10	0.36	0.24	0.93

^1^CON = control, 26 mL H_2_O/kg BW per 24 h; HGA = induced hindgut acidosis, 3 g wheat starch + 26 mL H_2_O/kg BW per 24 h.

^2^Trt = treatment effect; trt × time = treatment × time effect.

^3^In vitro dry matter digestibility.

### Ussing chamber

**Figure 5.**
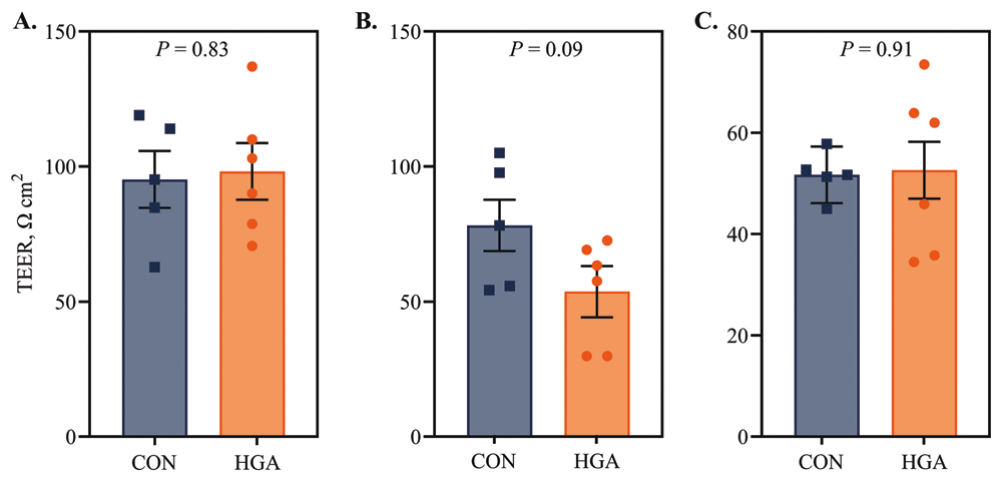
Effect of induced hindgut acidosis in ewes on transepithelial resistance (TEER) in ileum (A), cecum (B), and colon (C). CON = control, 26 mL H_2_O/kg BW per 24 h; HGA = induced hindgut acidosis, 3 g wheat starch + 26 mL H_2_O/kg BW per 24 h.

In cecal tissue, TEER tended (*P *= 0.09) to be lesser in HGA ewes than CON ewes (53.7 vs 78.2 ohms cm^2^, respectively), indicating increased barrier function (Figure 5). Transepithelial electrical resistance was not different (*P *≥ 0.83) between treatments in ileal or colonic tissues.

## Discussion

The current study utilized ruminally and cecally cannulated sheep as a novel experimental model of hindgut acidosis. Cecal cannulation facilitates direct induction and measurement of hindgut acidosis. Additionally, the model allowed for the investigation into the potential systemic effects of hindgut acidosis since acidosis induction was isolated to the cecum. The normal physiological pH of the hindgut in ruminants is above 6.0 (Wheeler and Noller, 1977). Given cecal pH was below 5.5 following hour 12 of the experiment, hindgut acidosis was successfully induced in HGA ewes through the cecal starch infusion. Diet type can affect cecal pH, with pH decreasing with greater dietary inclusion of NSC (Wheeler and Noller, 1977). Sheep fed diets containing either 60% or 80% cracked corn had cecal pH values ranging from 5.97 to 6.30 compared to 6.98 for those fed a 100% hay diet (Lewis and Dehority, 1985). Increasing the amount of substrate in the cecum through the infusion of wheat starch in the HGA ewes provided a greater amount of rapidly fermentable substrate for microbial fermentation in the hindgut. Previous research in sheep reported that cecal VFA concentrations increased with increasing amount of starch infused in the terminal ileum (Ørskov et al., 1970).

Fecal pH has been utilized as an indicator of hindgut acidosis in ruminants, but there is no clearly defined diagnostic threshold (Plaizier et al., 2018). After hour 12, fecal pH of the HGA ewes remained below 6 and reached a nadir of 5.06. This marked decrease in fecal pH in HGA ewes is further supportive of hindgut acidosis induction. Fecal pH in dairy cattle that received a 3 kg abomasal infusion of corn starch was 5.15 compared with 6.49 for control cows (van Gastelen et al., 2021a). Other studies induced hindgut acidosis with abomasal infusions of corn starch and reported nadir fecal pH values from 5.5 to 5.8 (Abeyta et al., 2023a,b,c). In this study, the similar trend in cecal and fecal pH suggests that fecal pH is a viable indicator of hindgut acidosis. Across both treatments, cecal pH was 0.3 units less than fecal pH on average. Thus, a fecal pH below 5.8 would indicate acidotic conditions in the hindgut and a pH below 5.5. Microbial fermentation in the hindgut is not limited to the cecum, but also occurs in the colon (Hoover, 1978). Increased dietary starch has been observed to increase VFA concentration in colonic digesta and feces (Tao et al., 2014; Neubauer et al., 2020). Additionally, fecal VFA concentrations were increased when hindgut acidosis was induced through abomasal starch infusions (van Gastelen et al., 2021b; Cronin et al., 2023). Therefore, increased fecal VFA concentration in the HGA ewes indicates a continued increase in fermentation in the cecum and colon due to increased substrate available for fermentation.

Fecal consistency changes in response to increased hindgut fermentation (Gressley et al., 2011). In the current study, HGA had decreased fecal DM compared with CON ewes despite receiving a similar infusion rate of H_2_0. This is consistent with decreased fecal consistency scores in dairy cattle with induced hindgut acidosis (van Gastelen et al., 2021a; Abeyta et al., 2023b). Increased solutes such as VFA and lactate increase the osmolality of rumen fluid, leading to an influx of water from the blood during ruminal acidosis (Owens et al., 1998). Increased VFA concentration in the hindgut due to increased microbial fermentation can also result in hyperosmolality of cecal contents (Mainardi et al., 2011). Thus, the decreased DM of feces in hindgut acidosis may result from the osmotic effects of acidotic cecal contents. Conversely, CON ewes had drier feces than HGA ewes and a greater volume of urine output despite receiving the same volume of water via cecal infusion. The colon has a regulatory role in water and electrolyte homeostasis in sheep (Meintjes and Engelbrecht, 1995). The amount of water that is reabsorbed in the colon is altered in accordance with an animal’s requirements (Schultz, 1984). Although water intake was not measured, total water output (urine + fecal water) did not differ between treatments, suggesting that overall water balance was maintained. Therefore, since osmolality was likely not altered in their hindgut due to induced acidosis, CON ewes likely reabsorbed the cecally infused water in the colon, resulting in drier feces and greater urinary output.

Mineral blocks were available during the experiment. Although mineral consumption was not measured, minimal consumption of the mineral blocks was noted during the short 4-d experiment. The blocks contained calcium carbonate and magnesium oxide, which could have affected buffering capacity in the rumen or hindgut. While it seems unlikely that mineral block consumption contributed to treatment differences in ruminal fermentation, this possibility cannot be entirely excluded.

In ruminants, fiber digestion occurs in both the rumen and the hindgut. The hindgut specifically can account for up to 27% of cellulose digested in the ruminant gastrointestinal tract (Ulyatt and Macrae, 1974; Hoover, 1978). Induced hindgut acidosis can decrease apparent total tract NDF digestion by 4% units in dairy cattle (van Gastelen et al., 2021a, b). Cellulolytic bacteria are inhibited at a pH below 5.8, which can negatively impact fiber digestion (Russell and Wilson, 1996). Cecal pH was not greater than 6.0 after hour 12 of the experiment for HGA ewes. Thus, depressed pH in the hindgut of HGA ewes would likely contribute to reduced total tract NDF and acid detergent fiber digestion.

To the best of the authors’ knowledge, this was the first study that sought to determine the effects of induced hindgut acidosis on rumen conditions. Rumen VFA concentration was greater while rumen pH was lesser in HGA ewes than in CON ewes. Dietary factors would not account for these observed differences, as the diet was the same between treatments and feed intake was controlled for all ewes. However, water intake and rumen volume were not measured during this experiment. Differences in liquid rumen pool size can impact VFA concentrations (Hall et al., 2015). Thus, a decrease in water intake or smaller rumen pool size in the HGA ewes could have resulted in greater total VFA concentration compared with CON ewes. There were no differences between treatments in rumen ex vivo fermentation evaluated on day 4. The lack of differences for the ex vivo fermentation could be due to the timing of rumen fluid collection. At hour 72, when rumen fluid was collected for use in the ex vivo model, rumen pH and total VFA concentration were not different between treatments. Additionally, given the reduction in fecal DM, it is possible that a shift of water from the rumen to the hindgut occurred in HGA ewes, which may have concentrated rumen contents and contributed to the observed differences in VFA concentration. Furthermore, the absence of differences in the ex vivo rumen fermentation suggests that rumen microbial activity may not have been directly altered by hindgut acidosis. While these data do not allow for definitive conclusions about changes in rumen fermentation, they do suggest that hindgut acidosis may have systemic effects that influence rumen environment or fluid dynamics. Systemic effects such as inflammation have been observed to disrupt ruminal metabolism. Dairy cattle that received an intravenous infusion of bacterial lipopolysaccharide had decreased rumen pH and increased rumen VFA concentration compared with those that received saline (Jing et al., 2014). Damage to the intestinal barrier can result in translocation of antigens and endotoxins into systemic circulation, leading to an inflammatory response (Kvidera et al., 2017). Thus, conditions in the cecum from induced acidosis could have negatively impacted gut barrier function, resulting in systemic responses that in turn affected the rumen environment. Previous models of induced hindgut acidosis utilizing abomasal starch infusions have not elicited a systemic inflammatory response as measured by blood biomarkers in dairy cattle (van Gastelen et al., 2021a, b; Abeyta et al., 2023a,b,c; Piantoni et al., 2023). However, inflammatory biomarkers are nonspecific responses to immune activation, and comparisons are limited since previous studies did not characterize effects on gut permeability.

In vivo markers, CrDTA and sucralose, were utilized to quantify gut permeability. These markers are of similar size (384 and 398 Da, respectively) and are not degraded by microbes in the intestinal tract, which makes them suitable probes in measuring hindgut paracellular permeability (Schoultz and Keita, 2020). Increased concentration of paracellular probes in the blood or urine indicates disruption in intestinal barrier function (Bischoff et al., 2014). Previous studies in ruminants reported an increase in urinary Cr excretion when gut barrier dysfunction was induced through aspirin administration (Briggs et al., 2020, 2021). However, no differences were reported in urinary CrEDTA or sucralose excretion when measuring the effects of heat stress in dairy cattle on gut permeability (Ellett et al., 2024). Additionally, in hindgut acidosis models using abomasal starch infusions, there were no differences in urinary CoEDTA excretion or blood CrEDTA appearance (van Gastelen et al., 2021a; Sanz-Fernandez et al., 2024). In all of the aforementioned studies, it is important to note that CrEDTA, CoEDTA, or sucralose were dosed either orally or abomasally, and gut permeability corresponded to the entire gastrointestinal tract. In the current study, markers were infused directly in the cecum to measure permeability specific to the hindgut specifically. For both CrEDTA and sucralose, no statistical differences were observed between CON and HGA ewes. However, the numerical difference between treatments of mesenteric plasma concentration of sucralose at hour 0.5 post-sucralose infusion is likely biologically relevant. To ensure differences in sucralose plasma concentration were not due to variation in mesenteric blood flow, PAH was used to estimate blood flow and was unaffected by treatment, time, or their interaction. Thus, the observed sucralose concentrations likely reflect differences in epithelial permeability rather than perfusion or blood flow rate. Notably, individual animal responses in in vivo markers of gut permeability were greatly variable.

The ex vivo measure of gut permeability in the Ussing chambers supports that hindgut acidosis negatively impacts gut barrier function. Ewes on the HGA treatment had a ~30% reduction in cecal TEER compared with CON ewes. While there is limited data regarding TEER in the ovine hindgut, this reduction is likely physiologically relevant. In mice, dextran sodium sulphate-induced colitis reduced colonic TEER by approximately 20% (Thomson et al., 2019). Colonic TEER was also reduced by ~20% in growing pigs that experienced heat stress (Pearce et al., 2012). In this study, there were no differences between HGA and CON ewes in TEER of the spiral colon. The lack of effect on colonic TEER, despite reductions observed in cecal TEER, may be due to the direct application of the HGA treatment to the cecum, resulting in more immediate and localized acidotic stress in that region. While fecal pH was decreased and fecal VFA concentrations were increased in HGA ewes, indicating that some degree of acidosis likely occurred in the colon, the intensity or duration of exposure may have been insufficient to cause measurable disruption to colonic TEER. Additionally, TEER measurements reflect only a small section of tissue and may not fully capture patchy or region-specific damage across the entire colonic surface. For this reason, multiple approaches were used to assess gut permeability in this study.

The gut permeability observations in the current study suggest that individual animal susceptibility to changes in intestinal barrier function varies widely. Despite the similar severity of induced hindgut acidosis in HGA ewes, the observed responses in gut permeability were less consistent. A variety of stressors can alter gut permeability (Lambert, 2009). Thus, control animals could have also experienced some disruptions in gut barrier function as a result of generalized stress from the experiment or unintentional damage to the cecal epithelium when sampling. Transit time in the hindgut was not quantified in the current study. However, intestinal motility and transit time differences could have potentially affected the absorption of in vivo markers of gut permeability and should be considered in future work (Odenwald and Turner, 2013). Measuring gut permeability is a complex task due to the numerous connections between the intestinal tract and other body systems (González-González et al., 2019). A greater understanding of the factors that contribute to intestinal barrier function in ruminants is needed.

The reduction in cecal TEER and the numerical increase in mesenteric plasma sucralose concentration in HGA ewes suggest that hindgut acidosis may impair epithelial barrier integrity. The gut epithelium functions as a critical barrier between intestinal contents and the systemic circulation. When this barrier is compromised, bacteria may translocate across the mucosa, contributing to extraintestinal infections (Berg, 1999). While ruminal barrier damage is commonly implicated in the pathogenesis of liver abscesses in cattle (Nagaraja and Lechtenberg, 2007), evidence that liver abscess-associated bacteria can originate from both the rumen and hindgut (Fuerniss et al., 2022) suggests that similar disruptions in the hindgut epithelium could represent another potential route of pathogen entry. Thus, the current observations support the need for future studies investigating the role of hindgut acidosis and post-ruminal gut health in liver abscesses.

## Conclusion

This novel model of a cecal starch infusion was successful at inducing hindgut acidosis in sheep. Cecal and fecal pH values followed a similar trend in response to hindgut acidosis, suggesting fecal pH is a viable indicator of increased hindgut fermentation. Although the starch infusion was localized to the cecum and DM intake was consistent across treatments, HGA ewes had higher rumen VFA concentrations and slightly lower rumen pH. These changes occurred without corresponding differences in ex vivo fermentation, suggesting that systemic responses to hindgut acidosis may have influenced rumen conditions. Decreased pH values of the hindgut likely contributed to reduced fiber digestibility in HGA ewes. Cecal epithelial permeability was affected by hindgut acidosis and was most clearly evident in the ex vivo assessment in Ussing chambers. However, ileal and colonic tissue permeability was not impacted by induced hindgut acidosis. In vivo markers of paracellular gut permeability were highly variable between individual animals, regardless of the comparable fermentation responses to cecal starch infusion in HGA ewes. This suggests animals vary in their individual susceptibility to disruptions in intestinal barrier function. Acidotic conditions in the cecum affected rumen conditions, but further investigation into hindgut acidosis’s impacts on gut permeability in ruminants is warranted.

## Abbreviations

ADF: acid detergent fiber
BW: body weight
CP: crude protein
DM: dry matter
DMI: dry matter intake
EE: ether extract
IVDMD: in vitro dry matter digestibility
NDF: neutral detergent fiber
PAH: paraaminohippurate
OM: organic matter
TEER: transepithelial resistance
VFA: volatile fatty acids
